# Letheon Administered in a Case of Labor

**Published:** 1847-04

**Authors:** N. C. Keep

**Affiliations:** Boston


16. *The Letheon administered in a Case of Labor.*–(To the editor of the Boston Medical and Surgical Journal.)—On the 7th inst. I administered the vapor of ether in a case of natural labor. The patient was in good health, and in labor of her third child. Five and a half hours having elapsed from the commencement of labor, her pains, which had been light, but regular, becoming severe, the vapor of ether was inhaled by the *nose*, and exhaled by the mouth. The patient had no difficulty in taking the vapor in this manner from the reservoir, without any valvular apparatus.

In the course of twenty minutes four pains had occurred without suffering, the vapor of ether being administered between each pain. Consciousness was unimpaired and labor not retarded. Inhalation was then suspended, that a comparison might be made between the effective force of the throes with and without the vapor of ether. No material difference was detected, but the distress of the patient was great. Inhalation was resumed, but the progress of the labor was so rapid that time could not be found for sufficient inhalation to bring the system *perfectly* under its influence; still the sufferings of the last moments were greatly mitigated. From the commencement of the inhalation to the close of the labor, thirty minutes. Number of inhalations, five. No unpleasant symptoms occurred, and the result was highly satisfactory.

Yours, &c.

*Boston*, *April* 10*th*, 1847. N. C. KEEP.

